# Particulate Matter Exposure and Cardiopulmonary Differences in the Multi-Ethnic Study of Atherosclerosis

**DOI:** 10.1289/ehp.1409451

**Published:** 2016-02-09

**Authors:** Carrie P. Aaron, Yana Chervona, Steven M. Kawut, Ana V. Diez Roux, Mingwu Shen, David A. Bluemke, Victor C. Van Hee, Joel D. Kaufman, R. Graham Barr

**Affiliations:** 1 Department of Medicine, College of Physicians and Surgeons, Columbia University, New York, New York, USA; 2 Department of Environmental Medicine, New York University, New York, New York, USA; 3 Department of Medicine, University of Pennsylvania School of Medicine, Philadelphia, Pennsylvania, USA; 4 Department of Epidemiology, School of Public Health, University of Michigan, Ann Arbor, Michigan, USA; 5 Radiology and Imaging Sciences, Clinical Center, National Institutes of Health, Department of Health and Human Services, Bethesda, Maryland, USA; 6 Department of Environmental and Occupational Health Sciences; 7 Department of Medicine, and; 8 Department of Epidemiology, University of Washington, Seattle, Washington, USA; 9 Department of Epidemiology, Mailman School of Public Health, Columbia University, New York, New York, USA

## Abstract

**Background:**

Particulate matter (PM) exposure may directly affect the pulmonary vasculature. Although the pulmonary vasculature is not easily measurable, differential associations for right ventricular (RV) and left ventricular (LV) mass may provide an indirect assessment of pulmonary vascular damage.

**Objectives:**

We tested whether long-term exposure to PM < 2.5 μm (PM2.5) is associated with greater RV mass and RV mass/end-diastolic volume ratio relative to the LV.

**Methods:**

The Multi-Ethnic Study of Atherosclerosis performed cardiac magnetic resonance (CMR) imaging among participants 45–84 years old without clinical cardiovascular disease in 2000–2002 in six U.S. cities. A fine-scale spatiotemporal model estimated ambient PM2.5 exposure in the year before CMR; individually weighted estimates accounted for indoor exposure to ambient PM2.5. Linear regression models were adjusted for demographics, anthropometrics, smoking status, cardiac risk factors, and LV parameters, with additional adjustment for city.

**Results:**

The 4,041 included participants had a mean age of 61.5 years, and 47% were never smokers. The mean ambient PM2.5 was 16.4 μg/m3 and individually weighted PM2.5 was 11.0 μg/m3. PM2.5 exposure was associated with greater RV mass [ambient: 0.11 g per 5 μg/m3 (95% CI: –0.05, 0.27); individually weighted: 0.20 g per 5 μg/m3 (95% CI: 0.04, 0.36)] and a greater RV mass/end-diastolic volume ratio conditional on LV parameters. City-adjusted results for RV mass were of greater magnitude and were statistically significant for both measures of PM2.5, whereas those for RV mass/end-diastolic volume ratio were attenuated.

**Conclusions:**

Long-term PM2.5 exposures were associated with greater RV mass and RV mass/end-diastolic volume ratio conditional on the LV; however, additional adjustment for city attenuated the RV mass/end-diastolic volume findings. These findings suggest that PM2.5 exposure may be associated with subclinical cardiopulmonary differences in this general population sample.

**Citation:**

Aaron CP, Chervona Y, Kawut SM, Diez Roux AV, Shen M, Bluemke DA, Van Hee VC, Kaufman JD, Barr RG. 2016. Particulate matter exposure and cardiopulmonary differences in the Multi-Ethnic Study of Atherosclerosis. Environ Health Perspect 124:1166–1173; http://dx.doi.org/10.1289/ehp.1409451

## Introduction

Exposure to ambient particulate matter (PM) has been linked to the occurrence of cardiovascular events (Brook et al. 2010; Pope et al. 2004). Although the causal mechanisms remain unclear, short- and long-term exposure to ambient PM has been associated with systemic endothelial dysfunction and a secondary inflammatory response in the vasculature (Krishnan et al. 2012; Nurkiewicz et al. 2004; Tamagawa et al. 2008). Animals exposed to PM for even short time periods have increased muscularization of pulmonary arterioles (Lemos et al. 2006; Rivero et al. 2005), suggesting increased pulmonary arteriolar pressure; however, it is unknown whether PM exposure has a similar effect in humans.

Although direct measurement of the pulmonary vasculature is not feasible in large epidemiologic studies, evaluation of cardiac structure can be used to indirectly assess chronic pulmonary vascular differences. We hypothesized that PM damages the pulmonary microvasculature, causing two distinct effects: increased right ventricular (RV) mass as a result of elevated pulmonary artery pressure (Bogaard et al. 2009) and reduced blood flow to the left ventricle (LV), resulting in LV underfilling and reduced stroke work (due to Starling’s Law), with a consequent reduction of LV mass and myocyte atrophy (Hardziyenka et al. 2011). This process would be analogous to pulmonary capillary damage in emphysema, which may increase RV mass and reduce LV end-diastolic volume and LV mass (Vonk Noordegraaf et al. 1997; Vonk Noordegraaf et al. 2005), and in pulmonary hypertension, in which the ratio of RV to LV mass on cardiac magnetic resonance (CMR) images predicts pulmonary artery pressure (Saba et al. 2002; Swift et al. 2013). Hence, we consider the association of an exposure with RV mass after adjustment for LV mass as the best surrogate for pulmonary vascular damage (see Figure S1).

Published results from the Multi-Ethnic Study of Atherosclerosis (MESA) indicate that long-term traffic-related air pollution exposure, assessed by nitrogen dioxide (NO_2_) levels, was associated with a greater RV mass after adjustment for LV mass (Leary et al. 2014), and that exposure to PM < 2.5 μm in diameter (PM_2.5_) was associated with a lower LV mass in analyses without adjustment for city (Van Hee et al. 2009). However, whether differences in RV relative to LV mass, which may reflect pulmonary vascular damage and related cardiopulmonary differences, occur in humans in relation to long-term PM exposure has not been assessed. We therefore examined the relationships between PM_2.5_ exposure and RV structure compared with LV structure on CMR in MESA, a large multi-ethnic cohort study. We hypothesized that greater PM_2.5_ exposure would be associated with increased RV mass and mass/end-diastolic volume ratio conditional on the LV.

## Methods

### Multi-Ethnic Study of Atherosclerosis

MESA is a multi-center prospective cohort study designed to investigate the prevalence, correlates, and progression of subclinical cardiovascular disease in whites, Hispanics, and African and Chinese Americans (Bild et al. 2002). In 2000–2002, MESA recruited 6,814 participants 45–84 years old from six U.S. communities. Multiple racial/ethnic groups were recruited at all sites to reduce site-by-race confounding. Exclusion criteria included clinical cardiovascular disease, weight over 300 lbs. (136.4 kg), pregnancy, or other impediment to long-term participation. The MESA Air Pollution Study was a large ancillary study funded by the U.S. Environmental Protection Agency (EPA) to add air pollution exposure assessments for each participant (Kaufman et al. 2012). The MESA-RV Study was an ancillary study funded by the National Heart, Lung, and Blood Institute (NHLBI) to characterize RV structure and function by CMR in the MESA population. The protocols of MESA and all studies described herein were approved by the Institutional Review Boards of all collaborating institutions and the NHLBI. All participants provided written informed consent.

### Cardiac Magnetic Resonance Imaging

Participants underwent CMR in 2000–2002, as previously described (Natori et al. 2006). All imaging was performed using 1.5 T magnets with electrocardiographic gating. Methods for interpretation of LV and RV parameters have been previously reported (Bluemke et al. 2008; Chahal et al. 2010).

Briefly, all RV image analysis was performed at one site by two independent analysts who used Windows workstations with QMASS software (Medis). The endocardial and epicardial borders of the RV were traced manually on short axis cine images at end-systole and end-diastole. RV end-diastolic volume and end-systolic volume were calculated using Simpson’s rule. RV mass was determined at end-diastole as the difference between the epicardial and endocardial volumes multiplied by the specific gravity of myocardium (1.05 g/mL) (Natori et al. 2006). RV stroke volume was calculated by subtracting RV end-systolic volume from end-diastolic volume. RV ejection fraction was calculated as RV stroke volume divided by end-diastolic volume. The intrareader intraclass correlation coefficients (ICCs) were 0.89–0.99, and the interreader ICCs from random blinded rereads were 0.80–0.96 for RV mass, end-diastolic volume, and ejection fraction (Kawut et al. 2011).

### PM_2.5_ Exposure Estimates

**Figure 1:**
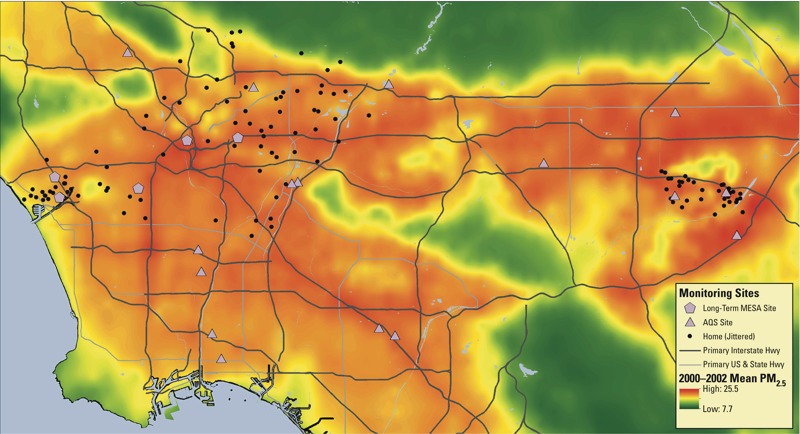
Map of 2000–2002 mean outdoor residential particulate matter < 2.5 μm in diameter (PM_2.5_) concentrations for Los Angeles Basin, CA, including the location of stationary monitoring sites operated by the South Coast Air Quality Management District (“AQS”), fixed sites operated by the MESA Air Study, and MESA Air participants’ homes where monitoring was conducted (jittered to protect privacy).

The MESA Air Pollution Study generated prediction models of long-term exposure to ambient PM_2.5_ based on each participant’s reported home address starting 1 year prior to the participant visit in 2000–2002 (Kaufman et al. 2012). The maximum likelihood predictions incorporated spatiotemporal modeling, which has been described previously (Keller et al. 2015; Sampson et al. 2011). Briefly, the model leveraged all available PM_2.5_ concentrations collected from the U.S. EPA’s Air Quality System monitors, one to five supplemental stationary monitors within each city, and monitoring at the homes of a subset of MESA participants (Cohen et al. 2009). The model also included geographic variables such as land use (e.g., industrial, residential, water), distance to various features including airports and coastlines, traffic volumes incorporated via dispersion models, and population density and urban topography (Cohen et al. 2009; Keller et al. 2015). Using concentrations predicted in 2-week averages at each participant’s home, we computed the annual average concentrations in the year before the first study visit and used this measure as a proxy of long-term exposure. Estimated exposures for ambient PM_2.5_ in Los Angeles are shown in Figure 1.

A secondary exposure, individually weighted PM_2.5_, was estimated using reported time spent indoors and the estimated infiltration fraction of ambient PM_2.5_. The infiltration fraction of ambient PM_2.5_ was estimated based on indoor and outdoor measurements of PM_2.5_, which were obtained at a small sample of participants’ homes using particulate sulfur as a tracer of outdoor particulates, and models incorporating home characteristics and behaviors (Allen et al. 2012; Kaufman et al. 2012; Spalt et al. 2015). These variables required completion of a home characteristics questionnaire at a follow-up visit in 2006–2008; thus, individually weighted PM_2.5_ is available only for a subset of study participants.

Estimates for PM_2.5_ exposures were weighted for time at each address if participants moved during the year.

### Covariate Information

Age, sex, race/ethnicity, educational attainment, income, smoking status, pack-years of smoking, and medical history were self-reported in 2000–2002. Height, weight, resting blood pressure, fasting serum glucose, C-reactive protein, total cholesterol, and high-density lipoproteins (HDL) were measured using standard techniques (MESA Manual of Operations: Field Center and Laboratory Procedures 2008). Hypertension was defined as blood pressure ≥ 140/90 mmHg or self-reported hypertension and use of antihypertensive medications. Diabetes was defined as fasting glucose ≥ 7.0 mmol/L (≥ 126 mg/dL), use of hypoglycemic medication, or self-reported physician diagnosis. Current smoking status was verified using a urinary cotinine assay (Rodriguez et al. 2010). Participant questionnaires included self-report of trouble breathing at night and of the intensity and duration of typical physical activity, which was quantified as metabolic equivalent (MET) minutes per week (Bertoni et al. 2009). The neighborhood socioeconomic status (SES) index represented six U.S. Census variables identified as unique contributors to neighborhood SES (Hajat et al. 2013). Ambient NO_2_ exposures were estimated using a model similar to that described for PM_2.5_ (Keller et al. 2015) and were weighted for time at each address if the participant moved.

Trained readers performed percent emphysema measurements on cardiac CT scans obtained between 2000 and 2002 using modified Pulmonary Analysis Software Suite (PASS) software (Hoffman et al. 2009). Percent emphysema was defined as the percentage of voxels in the lung below –950 Hounsfield units, adjusted for the attenuation of air outside the chest. Emphysema was defined as percent emphysema above the upper limit of normal calculated using reference equations (Hoffman et al. 2014). Spirometry was performed on a subset of participants between 2004 and 2007 in accordance with American Thoracic Society/European Respiratory Society guidelines (Miller et al. 2005) and following the MESA Lung protocol; all exams were reviewed by one investigator (Hankinson et al. 2010). Airflow limitation was defined as forced expiratory volume in 1 sec/forced vital capacity (FEV_1_/FVC) below 0.7.

### Statistical Analyses

The sample was stratified by quintile of ambient PM_2.5_ exposure for descriptive purposes; levels of categorical variables and means of continuous variables are shown in Table 1. Linear regression models were used to estimate the associations between PM exposures and RV parameters conditional on LV parameters. Adjustment for the corresponding LV parameter was performed to indirectly assess pulmonary vascular differences (see Figure S1). LV adjustment also accounted for other potential associations of LV with RV structure (e.g., greater LV mass causing elevated LV end-diastolic pressure leading to pulmonary venous hypertension and greater RV mass) and reduced confounding related to body size. Additionally, the multivariable model was adjusted for parameters thought to be associated with RV mass *a priori* (age, sex, race/ethnicity, height, weight, smoking status, pack-years, total cholesterol, HDL, hypertension, systolic blood pressure, fasting glucose, diabetes, and C-reactive protein) and confounders of air pollution exposure (education, income, neighborhood SES index). We present these multivariable models before and after adjustment for city, the latter being treated as the primary analysis in order to address unmeasured confounding by study site. Linear relationships were confirmed in generalized additive models by visual inspection (data not shown). We present all results and 95% confidence intervals (CIs), as recommended by Rothman (1990). In the primary analysis, effect modification of the PM and RV mass association on an additive scale was assessed using interaction terms by sex, race/ethnicity, age (above and below 60 years), smoking status (ever or never smoker), airflow limitation (yes/no), emphysema (yes/no) and city. Sensitivity analyses were performed limiting the sample to those who had lived at the same residence for more than 5 years before the study visit and adjusting for factors that may be associated with RV function or exposure to PM including percent emphysema, lung function (FEV_1_, FEV_1_/FVC), reported trouble breathing at night, and self-reported physical activity (with the exception of percent emphysema, these variables were available only for a subset of participants). Sensitivity analyses were also performed adjusting for NO_2_ exposure and using a random intercept for city. Analyses were performed in SAS 9.3 (SAS Institute Inc., Cary, NC).

**Table 1:** Characteristics of MESA participants with right and left ventricular parameters on cardiac magnetic resonance imaging and air pollution estimates in 2000–2002, by quintile of ambient PM_2.5_ exposure (*n* = 4,041).

Characteristic	Q1 (*n* = 808)	Q2 (*n* = 808)	Q3 (*n* = 809)	Q4 (*n* = 808)	Q5 (*n* = 808)
Age, years	60.5 ± 10.2	61.7 ± 10.1	61.8 ± 9.9	61.4 ± 9.8	62.3 ± 10.3
Male, %	50.1	50.2	44.5	42.2	51.1
Race, %
White	51.1	41.7	46.0	41.1	14.7
African American	13.0	43.4	36.1	30.2	12.0
Hispanic	32.4	9.3	11.5	22.5	33.4
Chinese American	3.5	5.6	6.4	6.2	39.9
Educational attainment, %^*a*^
Incomplete high school	13.9	11.5	11.1	17.0	26.5
Complete high school	20.9	17.8	18.8	16.1	19.2
Some college	33.0	29.3	29.4	26.2	24.3
Complete college	16.7	18.7	18.7	17.9	17.9
Graduate school	15.3	22.0	21.6	22.3	12.1
Gross family income, %^*b*^
Below $12,000	9.3	7.6	8.8	9.4	15.5
$12,000–$24,999	15.4	13.6	14.6	19.2	30.9
$25,000–$34,999	13.7	10.8	12.4	15.5	14.4
$35,000–$49,999	18.3	16.3	16.7	14.6	12.5
$50,000–$99,999	29.5	31.3	29.5	23.6	14.7
≥ $100,000	10.5	14.4	14.8	15.1	10.6
Neighborhood SES index^*c*^	–0.8 ± 4.3	–1.4 ± 5.7	–1.0 ± 6.0	–1.6 ± 7.6	–0.9 ± 7.1
Height, cm	166.9 ± 9.9	168.1 ± 10.2	167.0 ± 9.8	166.1 ± 9.7	164.1 ± 9.5
Weight, kg	79.7 ± 15.1	80.0 ± 16.5	78.8 ± 16.8	77.8 ± 16.0	71.7 ± 15.4
Body mass index, kg/m^2^	28.5 ± 4.8	28.2 ± 5.0	28.1 ± 5.0	28.1 ± 5.2	26.4 ± 4.6
Smoking, %
Never	44.2	46.2	42.2	44.9	56.4
Former	41.6	39.2	42.9	40.8	33.0
Current	14.2	14.5	15.0	14.2	10.5
Pack-years^*d*^	23.4 ± 23.7	23.9 ± 23.2	25.5 ± 25.9	26.1 ± 26.3	22.2 ± 22.7
Diabetes, %^*e*^	11.0	9.3	12.4	11.0	14.2
Fasting glucose, mg/dL	101.6 ± 26.2	101.8 ± 22.9	102.9 ± 29.3	102.2 ± 29.0	108.2 ± 35.6
Hypertension, %^*f*^	35.5	47.0	44.7	46.3	42.6
Systolic blood pressure, mmHg	122.3 ± 20.0	127.8 ± 21.9	125.5 ± 20.4	126.4 ± 20.4	125.9 ± 22.0
U.S. City, %
Forsyth County, North Carolina	7.9	26.9	24.7	14.5	—
New York, New York	4.2	21.0	24.4	43.8	7.8
Baltimore, Maryland	11.1	33.3	32.3	14.2	—
St. Paul, Minnesota	71.9	3.2	—	—	—
Chicago, Illinois	4.8	15.6	18.7	27.0	2.7
Los Angeles, California	—	—	—	0.5	89.5
C-reactive protein, mg/L	3.4 ± 4.9	3.6 ± 5.5	3.6 ± 5.4	4.0 ± 6.7	3.1 ± 5.5
HDL cholesterol, mg/dL	49.8 ± 14.9	50.9 ± 14.7	52.1 ± 15.7	53.3 ± 15.7	49.9 ± 14.2
Total cholesterol, mg/dL	198.9 ± 37.2	191.7 ± 35.2	195.1 ± 33.1	193.1 ± 35.0	192.5 ± 34.4
FEV_1_, L ^*g*^	2.65 ± 0.75	2.40 ± 0.69	2.37 ± 0.73	2.31 ± 0.70	2.37 ± 0.72
FEV_1_/FVC ratio^*h*^	0.75 ± 0.08	0.75 ± 0.08	0.75 ± 0.09	0.74 ± 0.09	0.75 ± 0.08
Airflow limitation, %^*h,i*^	19.0	22.0	24.2	24.8	20.2
Percent emphysema-950, median (IQR)^*j*^	2.37 (1.09, 4.64)	2.87 (1.28, 5.71)	2.84 (1.24, 5.59)	3.00 (1.27, 6.03)	3.43 (1.46, 6.26)
Emphysema above ULN, %^*k*^	8.5	9.6	8.1	6.9	4.5
Reported trouble breathing at night, %^*l*^	11.0	9.0	9.6	11.1	9.1
Reported physical activity, MET-min/week^*m*^	6,306 ± 5,508	5,246 ± 4,443	5,288 ± 4,455	5,358 ± 4,528	4,345 ± 4,531
Ambient PM_2.5_, μg/m^3^	12.5 ± 1.4	14.7 ± 0.4	15.7 ± 0.3	17.0 ± 0.6	22.1 ± 1.7
Individually weighted PM_2.5_, μg/m^3^	7.4 ± 1.5	9.2 ± 1.7	9.9 ± 1.9	11.4 ± 2.0	16.5 ± 2.4
Ambient NO_2_, ppm	13.7 ± 3.9	17.8 ± 7.9	20.0 ± 8.4	25.8 ± 8.2	31.5 ± 4.7
Abbreviations: HDL, high density lipoprotein; FEV_1_, forced expiratory volume in 1 sec; FVC, forced vital capacity; IQR, interquartile range; MET, metabolic equivalent of task; PM_2.5_, particulate matter ≤ 2.5 μm in diameter; SES, socioeconomic status; ULN, upper limit of normal. Values are the mean ± SD or %, except as noted. ^***a***^13 participants did not report educational attainment. ^***b***^134 participants did not report income. ^***c***^Higher numbers reflect greater SES. The neighborhood SES index combines several neighborhood SES variables (Hajat et al. 2013). ^***d***^Among 1,918 ever smokers; 233 did not report pack-years. ^***e***^Defined as fasting glucose ≥ 7.0 mmol/L (≥126 mg/dL) or hypoglycemic medication use. ^***f***^Defined as blood pressure ≥ 140/90 mmHg, or self-report and antihypertensive medication use. ^***g***^Among 2,657 spirometry grade A–D FEV_1_. ^***h***^Among 2,646 with spirometry grade A–D FEV_1_ and FVC. ^***i***^Defined as FEV_1_/FVC ratio below 0.7. ^***j***^Not available for one participant. ^***k***^ULN defined by reference equations (Hoffman et al. 2014); not available for 26 participants. ^***l***^33 participants did not report trouble breathing at night. ^***m***^209 participants did not report physical activity.					

## Results

MESA included 6,814 participants, of whom 5,098 underwent CMR with 5,004 being interpretable for the LV. Of the 4,634 participants selected for RV evaluation, reads were attempted in 4,484 before reaching a total of 4,204 interpretable scans (94% of attempted reads). PM_2.5_ exposure estimates were available for 4,057 of these participants, of whom 4,041 also had complete covariate data (see Figure S2). The 4,204 participants included in the MESA RV study did not differ from other MESA participants except that they were on average younger, had a lower BMI, and lower prevalence of diabetes and former and current smoking (Kawut et al. 2011). The mean (± SD) age of the sample was 61.5 ± 10 years, 52% were female, 47% were never smokers, 39% were white, 22% were Hispanic, 27% were African American, and 12% were Chinese American. The mean ambient PM_2.5_ exposure was 16.4 ± 3.4 μg/m^3^, and the mean individually weighted PM_2.5_ exposure was 11.0 ± 3.7 μg/m^3^. City-specific correlations of PM_2.5_ and NO_2_ exposures were moderate to high (0.53–0.81 for ambient and 0.32–0.55 for individually weighted PM_2.5_; see Table S1).

Compared with participants in the highest quintile of ambient PM_2.5_ exposure, those in the lowest quintile were more likely to be white and ever smokers; to have at least a high school education; greater height, weight, and FEV_1_; and a lower percent emphysema (Table 1). Participants in the lowest quintile were more likely to be in St. Paul, MN, whereas those in the highest quintile were more likely to be in Los Angeles, CA. RV mass, RV end-diastolic volume, stroke volume, LV mass, LV end-diastolic volume, and LV mass/end-diastolic volume ratio were greater, whereas RV mass/end-diastolic volume ratio and RV and LV ejection fraction were lower in the lowest quintile compared with the highest quintile of exposure (Table 2). The correlation between RV and LV mass was 0.62, the correlation between RV and end-diastolic volume was 0.82, the correlation between RV and stroke volume was 0.79, and the correlation between RV and ejection fraction was 0.47 (all *p*-values < 0.001).

**Table 2:** Cardiac magnetic resonance imaging parameters for participants with air pollution measures by quintile of ambient PM_2.5_ exposure (*n* = 4,041).

Imaging parameter	Q1 (*n *= 808)	Q2 (*n *= 808)	Q3 (*n *= 809)	Q4 (*n *= 808)	Q5 (*n *= 808)
RV
Mass, g	21.8 ± 4.8	21.2 ± 4.4	20.8 ± 4.4	21.1 ± 4.2	20.2 ± 4.1
End diastolic volume, mL	130.2 ± 33.5	125.9 ± 31.3	122.3 ± 29.5	124.5 ± 29.8	117.3 ± 28.2
Mass/end-diastolic volume ratio, g/mL	0.170 ± 0.02	0.171 ± 0.02	0.172 ± 0.02	0.172 ± 0.02	0.175 ± 0.02
Stroke volume, mL	90.4 ± 22.3	87.6 ± 20.2	86.2 ± 20.3	87.4 ± 20.2	82.3 ± 18.7
Ejection fraction, %	70.0 ± 6.4	70.2 ± 6.6	70.8 ± 6.6	70.6 ± 6.4	70.6 ± 6.2
LV
Mass, g	152.2 ± 38.4	148.2 ± 39.4	144.8 ± 37.8	147.0 ± 40.5	137.2 ± 38.4
End diastolic volume, mL	128.8 ± 31.6	126.3 ± 31.4	126.7 ± 32.3	128.5 ± 30.9	122.2 ± 30.2
Mass/end-diastolic volume ratio, g/mL	1.20 ± 0.25	1.19 ± 0.26	1.17 ± 0.27	1.16 ± 0.25	1.13 ± 0.20
Stroke volume, mL	86.5 ± 20.5	86.5 ± 19.9	86.2 ± 20.5	88.4 ± 19.5	84.6 ± 18.2
Ejection fraction, %	67.7 ± 7.6	69.1 ± 7.4	68.7 ± 7.5	69.4 ± 6.9	70.0 ± 7.5
Abbreviations: LV, left ventricle; RV, right ventricle. Values are the mean ± SD.					

### Ambient PM_2.5_ Exposure

Table 3 shows the associations of ambient PM_2.5_ with RV parameters conditional on LV parameters. In the multivariable model, ambient PM_2.5_ exposure was associated with a greater RV mass [0.11 g/5 μg/m^3^ (95% CI: –0.05, 0.27)] and mass/end-diastolic volume ratio and with a lower RV end-diastolic volume and stroke volume conditional on LV parameters. With adjustment for city, the relationship between PM_2.5_ and RV mass became stronger [0.37 g/5 μg/m^3^ (95% CI: 0.03, 0.71)], whereas associations with RV mass/end-diastolic volume ratio, end-diastolic volume and stroke volume were weakened (Table 3).

**Table 3:** Mean differences in RV mass, end-diastolic volume, mass/end-diastolic volume ratio, stroke volume and ejection fraction adjusted for LV parameters per 5 μg/m^3^ increase in ambient PM_2.5_ (*n *= 4,041) and individually weighted PM_2.5_ exposure (*n *= 3,379).

RV parameter adjusted for LV parameter	Ambient PM_2.5_ (95% CI)	Individually weighted PM_2.5_ (95% CI)
RV mass, g
Multivariable model	0.11 (–0.05, 0.27)	0.20 (0.04, 0.36)*
Multivariable model + city	0.37 (0.03, 0.71)*	0.30 (0.01, 0.59)*
RV end-diastolic volume, mL
Multivariable model	–2.57 (–3.38, –1.76)*	–1.51 (–2.32, –0.70)*
Multivariable model + city	–0.57 (–2.31, 1.17)	0.05 (–1.46, 1.56)
RV mass/end-diastolic volume ratio, g/mL
Multivariable model	0.003 (0.002, 0.004)*	0.002 (0.001, 0.003)*
Multivariable model + city	0.002 (–0.0002, 0.004)	0.001 (–0.001, 0.003)
Stroke volume, mL
Multivariable model	–2.20 (–2.78, –1.62)*	–1.20 (–1.80, –0.61)*
Multivariable model + city	–0.72 (–1.97, 0.54)	0.28 (–0.82, 1.39)
RV ejection fraction, %
Multivariable model	–0.28 (–0.57, –0.001)*	–0.11 (–0.40, 0.17)
Multivariable model + city	–0.18 (–0.80, 0.43)	0.15 (–0.39, 0.69)
Abbreviations: LV, left ventricle; PM_2.5_, particulate matter < 2.5 μm in diameter; RV, right ventricle. Multivariable model was adjusted for age, sex, race/ethnicity, height, weight, education, income, neighborhood SES index, smoking status, pack-years, total cholesterol, high-density lipoprotein, hypertension, systolic blood pressure, fasting glucose, diabetes, C-reactive protein, and respective left ventricular parameter. *p-Value < 0.05.		

**Figure 2:**
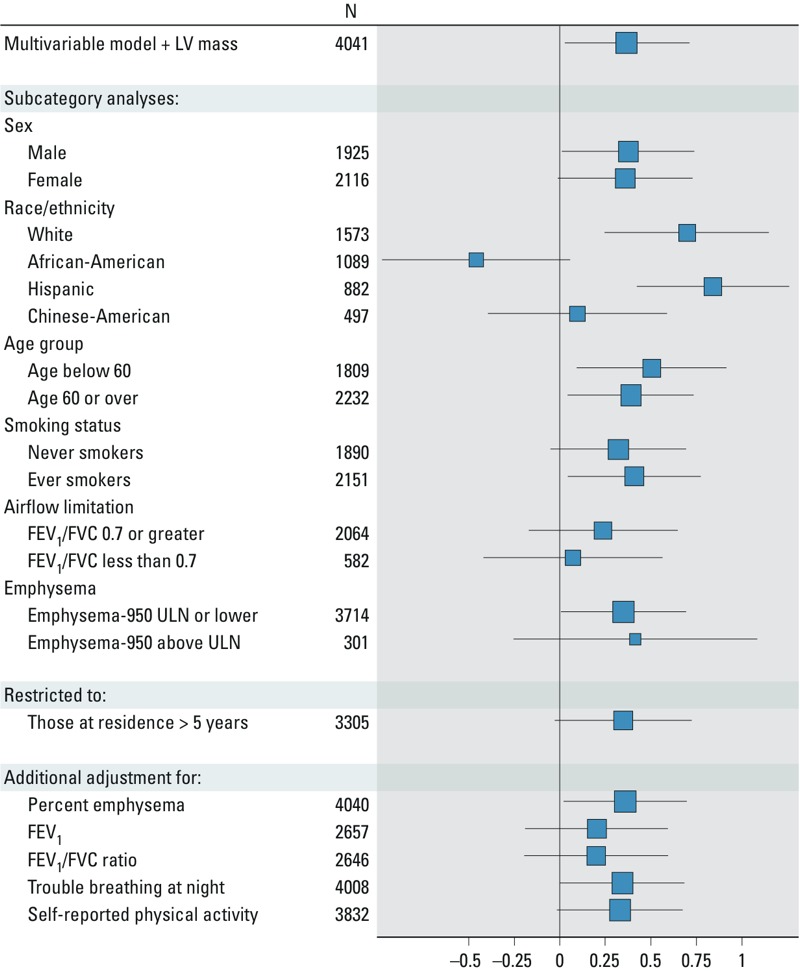
Sensitivity analyses for the multivariable association of ambient particulate matter < 2.5 μm in diameter (PM_2.5_) exposure and right ventricular (RV) mass adjusted for left ventricular (LV) mass and city. The mean differences (■) and 95% confidence intervals for a 5 μg/m^3^ change in PM_2.5_ are shown. The size of the square reflects the relative precision of the estimate; larger squares indicate greater precision. Multivariable model: adjusted for age, sex, race/ethnicity, height, weight, education, income, neighborhood socioeconomic status (SES) index, smoking status, pack-years, total cholesterol, high-density lipoprotein (HDL), hypertension, systolic blood pressure, fasting glucose, diabetes, C-reactive protein, LV mass and city. *p*-Interactions: sex, 0.91; race/ethnicity, 0.003; age group, 0.43; smoking status, 0.54; airflow limitation, 0.41; emphysema 0.84.

There was no evidence for statistically significant effect modification of the relationship between PM_2.5_ and RV mass conditional on LV mass by sex, age, smoking status, airflow limitation, or emphysema (Figure 2). However, there was significant interaction by race/ethnicity in the city-adjusted model (*p*-interaction = 0.003), and results were positive among whites [0.70 g/5 μg/m^3^ (95% CI: 0.25, 1.14)]and Hispanics [0.84 g/5 μg/m^3^ (95% CI: 0.42, 1.26)], and negative among African Americans [–0.46 g/5 μg/m^3^ (95% CI: –0.97, 0.06)] (Figure 2). Associations were largely unchanged when limited to the 82% who had been at the same residence for more than 5 years and after adjusting for percent emphysema, FEV_1_, FEV_1_/FVC, reported trouble breathing at night, or self-reported physical activity in the subsets with these measures (Figure 2).

There was significant effect modification for RV mass conditional on LV mass by city (*p*-interaction < 0.001) with large variation between the extremes of St. Paul, MN [3.86 g/5 μg/m^3^ (95% CI: 2.56, 5.16)] and Forsyth County, NC [–0.84 g/5 μg/m^3^ (95% CI: –2.20, 0.51)] (see Table S2). City-adjusted results were similar using a random intercept for city (see Table S3).

In models including NO_2_, the multivariable association between ambient PM_2.5_ and RV mass conditional on LV mass was in the opposite direction [–0.12 g/5 μg/m^3^ (95% CI: –0.32, 0.09)], and the city-adjusted association was attenuated [0.09 g/5 μg/m^3^ (95% CI: –0.34, 0.52)]. However, results were of a greater magnitude (with less precision) in the multivariable and city-adjusted models for RV mass/end-diastolic volume ratio, end-diastolic volume [multivariable model –4.01 mL/5 μg/m^3^ (95% CI: –5.05, –2.96), city-adjusted model –2.53 mL/5 μg/m^3^ (95% CI: –4.74, –0.32)], and stroke volume compared with the main results (see Table S4).

### Individually Weighted PM_2.5_ Exposure

Participants with measures of individually weighted PM_2.5_ did not appreciably differ from the overall sample (see Table S5). Individually weighted PM_2.5_ exposure was associated with greater RV mass [0.20 g/5 μg/m^3^ (95% CI: 0.04, 0.36)] and mass/end-diastolic volume ratio, as well as with lower end-diastolic volume [–1.51 mL/5 μg/m^3^ (95% CI: –2.32, –0.70)] and stroke volume conditional on LV parameters in the multivariable model (Table 3). The association for RV mass was of greater magnitude after adjustment for city [0.30 g/5 μg/m^3^ (95% CI: 0.01, 0.59)], whereas that of mass/end-diastolic volume ratio was attenuated, and associations for end-diastolic volume and stroke volume were no longer present (Table 3).

Sensitivity analyses for individually weighted PM_2.5_ are shown in Figure S3. Similarly to ambient PM_2.5_, there were significant interactions by race/ethnicity (with positive associations for whites and Hispanics and a negative association for African Americans), but no interaction by sex, age, smoking status, airflow limitation, or emphysema. For individually weighted PM_2.5_ there was also significant interaction of the association with RV mass by city (*p*-interaction < 0.001, data not shown).

Further adjustment for NO_2_ exposure resulted in attenuated associations between individually weighted PM_2.5_ and RV mass [multivariable model 0.04 g/5 μg/m^3^ (95% CI: –0.19, 0.26), city-adjusted model 0.21 g/5 μg/m^3^ (95% CI: –0.06, 0.47)]. Additionally, after adjustment for NO_2_ in the multivariable model, there were associations of greater magnitude and less precision for mass/end-diastolic volume ratio, end-diastolic volume [–2.66 mL/5 μg/m^3^ (95% CI: –3.80, –1.51)], and stroke volume, whereas city-adjusted results remained null (see Table S4).

## Discussion

In this study, we found that in a large cohort free of clinical cardiovascular disease, higher ambient and individually weighted PM_2.5_ exposures were associated with greater RV mass conditional on LV mass in models with and without adjustment for city. In addition, PM_2.5_ exposures were associated with greater RV mass/end-diastolic volume ratio and lower end-diastolic volume and stroke volumes conditional on LV parameters before adjustment for city. These findings provide evidence in the general population that PM_2.5_ exposure is associated with differences in cardiac structure, possibly reflecting pulmonary vascular differences.

Prior literature on this topic in humans is limited, likely because of difficulty in quantifying long-term individual exposures and in measuring the pulmonary vasculature. One study, which directly evaluated this relationship in 81 healthy children in Mexico, found that long-term ambient PM exposure was associated with increased mean pulmonary arterial pressure on transthoracic echocardiography and that acute PM exposures were associated with elevated serum endothelin (ET)-1 levels (Calderón-Garcidueñas et al. 2007).

In experimental settings, PM_2.5_ has various effects on the pulmonary vasculature including increased levels of vasoconstrictive proteins such as ET-1 (Matsumoto et al. 2010), pulmonary and systemic inflammation, oxidative stress, and platelet activation (Emmerechts et al. 2012; Marchini et al. 2014; Nurkiewicz et al. 2006). In animal studies, exposure to PM has been associated with reduced endothelial-derived vasodilation (Nurkiewicz et al. 2004; Tamagawa et al. 2008) and increased muscularization of pulmonary arterioles (Lemos et al. 2006; Rivero et al. 2005). Although some studies suggest that these phenomena also occur in humans (Delfino et al. 2009; Peretz et al. 2008; Zhang et al. 2013), it remains unclear whether they are due to local inflammation caused by inhaled particles or by particle translocation into the circulation.

Our finding of greater RV mass, conditional on LV mass, with greater PM_2.5_ exposure suggests adaptation to an elevated RV afterload (i.e., increased pulmonary vascular resistance). The associations between PM_2.5_ and increased mass/end-diastolic volume ratio and reduced RV end-diastolic volume and stroke volume are consistent with compensatory remodeling to lessen wall stress in response to increased pressures, as has been proposed to explain LV concentric remodeling in systemic hypertension (Ganau et al. 1992). Although the described changes in RV structure are small in magnitude (1–2% increase per 5 μg/m^3^), they may reflect important pulmonary vascular differences in this general population sample without significant cardiopulmonary disease.

We evaluated for confounding and effect modification by emphysema and airflow obstruction. Importantly, greater percent emphysema has been associated with a reduced proportion of small pulmonary vessels (Estépar et al. 2013), reduced RV and LV end-diastolic volume (Barr et al. 2010; Kawut et al. 2014), and increased PM_2.5_ exposure in this cohort (Adar et al. 2015). The observed associations were largely unchanged in those with and without emphysema and airflow obstruction and after adjusting for percent emphysema, FEV_1_, and FEV_1_/FVC.

Given the recently published association between NO_2_ exposure and increased RV mass and end-diastolic volume in MESA (Leary et al. 2014), we have presented results for PM_2.5_ adjusted for NO_2_ exposure. Findings for ambient PM_2.5_ and RV mass in the NO_2_ and city-adjusted model were attenuated (and were in the opposite direction in the multivariable model), but those for end-diastolic volume, stroke volume, and mass/end-diastolic volume ratio were of greater magnitude in the multivariable model. Because the correlations between 1-year ambient PM_2.5_ and NO_2_ exposures in this study were moderate to high (likely because of overlapping sources and similar modeling of exposure), and because NO_2_-adjusted results do not isolate PM-specific findings, these results should be interpreted cautiously.

The strengths of this study include the advanced PM_2.5_ exposure modeling for six cities across the United States, the use of CMR measures of ventricular structure and function, and the multi-ethnic general population sample. However, there are a number of limitations that should be discussed.

First, the city-specific results were highly variable. Adjustment for city was performed to account for potential unmeasured confounders, and these results are preferred. However, differences in both the levels of exposure and the variation in exposure by city may contribute to differences among the within-city estimates. In addition, the smaller sample size for each city may have led to unstable effect estimates within each city. Planned differences in the recruitment of racial/ethnic groups may have contributed to the effect modification seen by city (Hispanic participants were recruited in New York, St. Paul, and Los Angeles; Chinese Americans in Chicago and Los Angeles). Additionally, results varied by race/ethnicity with stronger direct associations between RV mass and PM_2.5_ for whites and Hispanics. Although these findings may be related to residual confounding by site, they should be evaluated further. Second, there is inevitably some misclassification of PM exposure. PM_2.5_ exposure was estimated using a complex spatiotemporal model to estimate exposure at each participant’s home, but exposures at other locations were not assessed. Our primary exposure of interest was ambient PM_2.5_ owing to potential measurement error in the variables used to estimate individually weighted PM_2.5_ (infiltration fraction and time spent indoors) and to the assumption that participants’ behaviors and home characteristics did not change significantly in the 5 years between measurement of end points and questionnaire completion. Although a small number of participants moved or retired during this period, the results were consistent for participants living at the same residence for at least 5 years. Exposure measurement error could affect our ability to make inferences, and beyond efforts to characterize exposure accurately, we did not correct for potential measurement error in this analysis (Sheppard et al. 2012). Third, we used CMR measurements of the RV and LV as proxies for the pulmonary vasculature; however, the ratio of RV to LV mass has been found to be a major predictor of mean pulmonary arterial pressure on right heart catheterization (Saba et al. 2002; Swift et al. 2013). Future studies may provide direct assessment of the pulmonary vasculature using recently developed noninvasive measures (Estépar et al. 2013; Hueper et al. 2013).

Because our findings are cross-sectional, reverse causality and selection bias must be considered. Reverse causality is unlikely because PM exposure is not plausibly altered by an individual’s cardiac structure. Selection bias is also unlikely because participants were recruited from the general population. Finally, although confounding is a concern in any observational study, we attempted to minimize residual confounding by adjusting for many factors, carefully measured in MESA, that can affect cardiac structure. Further studies to confirm these findings in longitudinal analyses and to evaluate potential mechanisms are warranted.

## Conclusion

Greater ambient and individually weighted ambient–derived PM_2.5_ exposures were associated with greater RV mass conditional on changes in the LV, and in non–city-adjusted models, with a greater RV mass/end-diastolic volume ratio. These findings suggest that PM_2.5_ exposure may contribute to subclinical pulmonary vascular differences in the general population.

## Supplemental Material

(926 KB) PDF

## References

[r1] Adar SD Kaufman JD Diez-Roux AV Hoffman EA D’Souza J Stukovsky KH 2015 Air pollution and percent emphysema identified by computed tomography in the Multi-Ethnic Study of Atherosclerosis. Environ Health Perspect 123 144 151, doi:10.1289/ehp.1307951 25302408 PMC4314244

[r2] Allen RW Adar SD Avol E Cohen M Curl CL Larson T 2012 Modeling the residential infiltration of outdoor PM_2.5_ in the Multi-Ethnic Study of Atherosclerosis and Air Pollution (MESA Air). Environ Health Perspect 120 824 830, doi:10.1289/ehp.1104447 22534026 PMC3385439

[r3] Barr RG, Bluemke DA, Ahmed FS, Carr JJ, Enright PL, Hoffman EA (2010). Percent emphysema, airflow obstruction, and impaired left ventricular filling.. N Engl J Med.

[r4] Bertoni AG, Whitt-Glover MC, Chung H, Le KY, Barr RG, Mahesh M (2009). The association between physical activity and subclinical atherosclerosis: the Multi-Ethnic Study of Atherosclerosis.. Am J Epidemiol.

[r5] Bild DE, Bluemke DA, Burke GL, Detrano R, Diez Roux AV, Folsom AR (2002). Multi-Ethnic Study of Atherosclerosis: objectives and design.. Am J Epidemiol.

[r6] Bluemke DA, Kronmal RA, Lima JA, Liu K, Olson J, Burke GL (2008). The relationship of left ventricular mass and geometry to incident cardiovascular events: the MESA (Multi-Ethnic Study of Atherosclerosis) study.. J Am Coll Cardiol.

[r7] Bogaard HJ, Abe K, Vonk Noordegraaf A, Voelkel NF (2009). The right ventricle under pressure: cellular and molecular mechanisms of right-heart failure in pulmonary hypertension.. Chest.

[r8] Brook RD, Rajagopalan S, Pope CA, Brook JR, Bhatnagar A, Diez-Roux AV (2010). Particulate matter air pollution and cardiovascular disease: an update to the scientific statement from the American Heart Association.. Circulation.

[r9] Calderón-Garcidueñas L Vincent R Mora-Tiscareño A Franco-Lira M Henríquez-Roldán C Barragán-Mejía G 2007 Elevated plasma endothelin-1 and pulmonary arterial pressure in children exposed to air pollution. Environ Health Perspect 115 1248 1253, doi:10.1289/ehp.9641 17687455 PMC1940106

[r10] Chahal H, Johnson C, Tandri H, Jain A, Hundley WG, Barr RG (2010). Relation of cardiovascular risk factors to right ventricular structure and function as determined by magnetic resonance imaging (results from the Multi-Ethnic Study of Atherosclerosis).. Am J Cardiol.

[r11] Cohen MA, Adar SD, Allen RW, Avol E, Curl CL, Gould T (2009). Approach to estimating participant pollutant exposures in the Multi-Ethnic Study of Atherosclerosis and Air Pollution (MESA Air).. Environ Sci Technol.

[r12] Delfino RJ Staimer N Tjoa T Gillen DL Polidori A Arhami M 2009 Air pollution exposures and circulating biomarkers of effect in a susceptible population: clues to potential causal component mixtures and mechanisms. Environ Health Perspect 117 1232 1238, doi:10.1289/ehp.0800194 19672402 PMC2721866

[r13] Emmerechts J, De Vooght V, Haenen S, Loyen S, van Kerckhoven S, Hemmeryckx B (2012). Thrombogenic changes in young and old mice upon subchronic exposure to air pollution in an urban roadside tunnel.. Thromb Haemost.

[r14] Estépar RS, Kinney GL, Black-Shinn JL, Bowler RP, Kindlmann GL, Ross JC (2013). Computed tomographic measures of pulmonary vascular morphology in smokers and their clinical implications.. Am J Respir Crit Care Med.

[r15] Ganau A, Devereux RB, Roman MJ, de Simone G, Pickering TG, Saba PS (1992). Patterns of left ventricular hypertrophy and geometric remodeling in essential hypertension.. J Am Coll Cardiol.

[r16] Hajat A Diez-Roux AV Adar SD Auchincloss AH Lovasi GS O’Neill MS 2013 Air pollution and individual and neighborhood socioeconomic status: evidence from the Multi-Ethnic Study of Atherosclerosis (MESA). Environ Health Perspect 121 1325 1333, doi:10.1289/ehp.1206337 24076625 PMC3855503

[r17] Hankinson JL, Kawut SM, Shahar E, Smith LJ, Stukovsky KH, Barr RG (2010). Performance of American Thoracic Society-recommended spirometry reference values in a multiethnic sample of adults: the Multi-Ethnic Study of Atherosclerosis (MESA) lung study.. Chest.

[r18] Hardziyenka M, Campian ME, Reesink HJ, Surie S, Bouma BJ, Groenink M (2011). Right ventricular failure following chronic pressure overload is associated with reduction in left ventricular mass: evidence for atrophic remodeling.. J Am Coll Cardiol.

[r19] Hoffman EA, Ahmed FS, Baumhauer H, Budoff M, Carr JJ, Kronmal R (2014). Variation in the percent of emphysema-like lung in a healthy, nonsmoking multiethnic sample. The MESA Lung Study.. Ann Am Thorac Soc.

[r20] Hoffman EA, Jiang R, Baumhauer H, Brooks MA, Carr JJ, Detrano R (2009). Reproducibility and validity of lung density measures from cardiac CT scans—the Multi-Ethnic Study of Atherosclerosis (MESA) Lung Study.. Acad Radiol.

[r21] Hueper K, Parikh MA, Prince MR, Schoenfeld C, Liu C, Bluemke DA (2013). Quantitative and semiquantitative measures of regional pulmonary microvascular perfusion by magnetic resonance imaging and their relationships to global lung perfusion and lung diffusing capacity: the Multiethnic Study of Atherosclerosis Chronic Obstructive Pulmonary Disease Study.. Invest Radiol.

[r22] Kaufman JD, Adar SD, Allen RW, Barr RG, Budoff MJ, Burke GL (2012). Prospective study of particulate air pollution exposures, subclinical atherosclerosis, and clinical cardiovascular disease: the Multi-Ethnic Study of Atherosclerosis and Air Pollution (MESA Air).. Am J Epidemiol.

[r23] Kawut SM, Lima JA, Barr RG, Chahal H, Jain A, Tandri H (2011). Sex and race differences in right ventricular structure and function: the Multi-Ethnic Study of Atherosclerosis–Right Ventricle Study.. Circulation.

[r24] Kawut SM, Poor HD, Parikh MA, Hueper K, Smith BM, Bluemke DA (2014). Cor pulmonale parvus in chronic obstructive pulmonary disease and emphysema: the MESA COPD study.. J Am Coll Cardiol.

[r25] Keller JP Olives C Kim SY Sheppard L Sampson PD Szpiro AA 2015 A unified spatiotemporal modeling approach for predicting concentrations of multiple air pollutants in the Multi-Ethnic Study of Atherosclerosis and Air Pollution. Environ Health Perspect 123 301 309, doi:10.1289/ehp.1408145 25398188 PMC4384200

[r26] Krishnan RM, Adar SD, Szpiro AA, Jorgensen NW, Van Hee VC, Barr RG (2012). Vascular responses to long- and short-term exposure to fine particulate matter: MESA Air (Multi-Ethnic Study of Atherosclerosis and Air Pollution).. J Am Coll Cardiol.

[r27] Leary PJ, Kaufman JD, Barr RG, Bluemke DA, Curl CL, Hough CL (2014). Traffic-related air pollution and the right ventricle. The Multi-Ethnic Study of Atherosclerosis.. Am J Respir Crit Care Med.

[r28] Lemos M, Mohallen SV, Macchione M, Dolhnikoff M, Assunção JV, Godleski JJ (2006). Chronic exposure to urban air pollution induces structural alterations in murine pulmonary and coronary arteries.. Inhal Toxicol.

[r29] Marchini T, Magnani ND, Paz ML, Vanasco V, Tasat D, González Maglio DH (2014). Time course of systemic oxidative stress and inflammatory response induced by an acute exposure to Residual Oil Fly Ash.. Toxicol Appl Pharmacol.

[r30] Matsumoto G, Nakagawa NK, Vieira RP, Mauad T, da Silva LF, de André CD (2010). The time course of vasoconstriction and endothelin receptor A expression in pulmonary arterioles of mice continuously exposed to ambient urban levels of air pollution.. Environ Res.

[r31] MESA Manual of Operations: Field Center and Laboratory Procedures (2008). Multi-Ethnic Study of Atherosclerosis Field Center Manual of Operations.. http://www.mesa-nhlbi.org/publicDocs/MesaMop/MesaMop1-5-01.doc.

[r32] Miller MR, Hankinson J, Brusasco V, Burgos F, Casaburi R, Coates A (2005). Standardisation of spirometry.. Eur Respir J.

[r33] Natori S, Lai S, Finn JP, Gomes AS, Hundley WG, Jerosch-Herold M (2006). Cardiovascular function in the Multi-Ethnic Study of Atherosclerosis: normal values by age, sex, and ethnicity.. AJR Am J Roentgenol.

[r34] Nurkiewicz TR Porter DW Barger M Castranova V Boegehold MA 2004 Particulate matter exposure impairs systemic microvascular endothelium-dependent dilation. Environ Health Perspect 112 1299 1306, doi:10.1289/ehp.7001 15345343 PMC1247520

[r35] Nurkiewicz TR Porter DW Barger M Millecchia L Rao KM Marvar PJ 2006 Systemic microvascular dysfunction and inflammation after pulmonary particulate matter exposure. Environ Health Perspect 114 412 419, doi:10.1289/ehp.8413 16507465 PMC1392236

[r36] Peretz A Sullivan JH Leotta DF Trenga CA Sands FN Allen J 2008 Diesel exhaust inhalation elicits acute vasoconstriction *in vivo*. Environ Health Perspectives 116 937 942, doi:10.1289/ehp.11027 PMC2453163 18629317

[r37] Pope CA, Burnett RT, Thurston GD, Thun MJ, Calle EE, Krewski D (2004). Cardiovascular mortality and long-term exposure to particulate air pollution: epidemiological evidence of general pathophysiological pathways of disease.. Circulation.

[r38] Rivero DH, Soares SR, Lorenzi-Filho G, Saiki M, Godleski JJ, Antonangelo L (2005). Acute cardiopulmonary alterations induced by fine particulate matter of São Paulo, Brazil.. Toxicol Sci.

[r39] Rodriguez J, Jiang R, Johnson WC, MacKenzie BA, Smith LJ, Barr RG (2010). The association of pipe and cigar use with cotinine levels, lung function, and airflow obstruction: a cross-sectional study.. Ann Intern Med.

[r40] Rothman KJ (1990). No adjustments are needed for multiple comparisons.. Epidemiology.

[r41] Saba TS, Foster J, Cockburn M, Cowan M, Peacock AJ (2002). Ventricular mass index using magnetic resonance imaging accurately estimates pulmonary artery pressure.. Eur Respir J.

[r42] Sampson PD, Szpiro AA, Sheppard L, Lindström J, Kaufman JD (2011). Pragmatic estimation of a spatio-temporal air quality model with irregular monitoring data.. Atmos Environ.

[r43] Sheppard L, Burnett RT, Szpiro AA, Kim SY, Jerrett M, Pope CA (2012). Confounding and exposure measurement error in air pollution epidemiology.. Air Qual Atmos Health.

[r44] Spalt EW, Curl CL, Allen RW, Cohen M, Adar SD, Hinckley Stukovsky KD, et al 2015 Time-location patterns of a diverse population of older adults: the Multi-Ethnic Study of Atherosclerosis and Air Pollution (MESA Air). J Expo Sci Environ Epidemiol doi:10.1038/jes.2015.29 PMC4641054 25921083

[r45] Swift AJ, Rajaram S, Hurdman J, Hill C, Davies C, Sproson TW (2013). Noninvasive estimation of PA pressure, flow, and resistance with CMR imaging: derivation and prospective validation study from the ASPIRE Registry.. JACC Cardiovasc Imaging.

[r46] Tamagawa E, Bai N, Morimoto K, Gray C, Mui T, Yatera K (2008). Particulate matter exposure induces persistent lung inflammation and endothelial dysfunction.. Am J Physiol Lung Cell Mol Physiol.

[r47] Van Hee VC, Adar SD, Szpiro AA, Barr RG, Bluemke DA, Diez Roux AV (2009). Exposure to traffic and left ventricular mass and function: the Multi-Ethnic Study of Atherosclerosis.. Am J Respir Crit Care Med.

[r48] Vonk Noordegraaf A, Marcus JT, Holverda S, Roseboom B, Postmus PE (2005). Early changes of cardiac structure and function in COPD patients with mild hypoxemia.. Chest.

[r49] Vonk Noordegraaf A, Marcus JT, Roseboom B, Postmus PE, Faes TJ, Vries PM (1997). The effect of right ventricular hypertrophy on left ventricular ejection fraction in pulmonary emphysema.. Chest.

[r50] Zhang J, Zhu T, Kipen H, Wang G, Huang W, Rich D (2013). Cardiorespiratory biomarker responses in healthy young adults to drastic air quality changes surrounding the 2008 Beijing Olympics.. Res Rep Health Eff Inst.

