# Supplementary material for: Particulate Matter Exposure and Cardiopulmonary Differences in the Multi-Ethnic Study of Atherosclerosis
Source: Environ Health Perspect. 2016 Feb 9;124(8):1166–73. doi: 10.1289/ehp.1409451 (PMC4977039; doi:10.1289/ehp.1409451)
Supplement: (926 KB) PDF [file ehp.1409451.s001.acco.pdf]

Environ Health Perspect
DOI: 10.1289/ehp.1409451

***Editor's Note**: In the original Supplemental Material, the legend for Figure S3 was incorrect. The size of the square does not reflect the relative number of participants in each group but instead reflects the relative precision of the estimate. Larger squares represent more precision. The figure legend has been corrected.*

Note to readers with disabilities: *EHP* strives to ensure that all journal content is accessible to all readers. However, some figures and Supplemental Material published in *EHP* articles may not conform to 508 standards due to the complexity of the information being presented. If you need assistance accessing journal content, please contact ehp508@niehs.nih.gov. Our staff will work with you to assess and meet your accessibility needs within 3 working days.

Supplemental Material

Particulate Matter Exposure and Cardiopulmonary Differences in the Multi-Ethnic Study of Atherosclerosis

Carrie P. Aaron, Yana Chervona, Steven M. Kawut, Ana V. Diez Roux, Mingwu Shen, David A. Bluemke, Victor C. Van Hee, Joel D. Kaufman, and R. Graham Barr

Table of Contents

Table S1: City-specific and overall correlations between $PM_{2.5}$ and NO_2 exposures

Table S2: Mean differences in RV mass, end-diastolic volume, mass/end-diastolic volume ratio, stroke volume and ejection fraction adjusted for LV parameters per 5 $\mu g/m^3$ increase in ambient $PM_{2.5}$ exposure, stratified by city

Table S3: Mean differences in RV mass, end-diastolic volume, mass/end-diastolic volume ratio, stroke volume and ejection fraction adjusted for LV parameters per 5 $\mu g/m^3$ increase in ambient $PM_{2.5}$ and individually-weighted $PM_{2.5}$ exposure, with a random effect for city

Table S4: Mean differences in RV mass, end-diastolic volume, mass/end-diastolic volume ratio, stroke volume and ejection fraction adjusted for LV parameters and NO_2 exposure per 5 $\mu g/m^3$ increase in ambient $PM_{2.5}$ (N=4,028) and individually-weighted $PM_{2.5}$ (N=3,368) exposure

Table S5: Characteristics of participants with ambient $PM_{2.5}$ measured and the subset with individually-weighted $PM_{2.5}$

Figure S1: Hypothesized causal diagram

Figure S2: Description of the study sample

Figure S3: Sensitivity analyses for the multivariable association of individually-weighted $PM_{2.5}$ exposure and RV mass adjusted for LV mass and city. Shown are the mean differences (■) and 95% confidence limits for a 5 μg/m^3 change in $PM_{2.5}$. The size of the square reflects the relative precision of the estimate. Larger squares represent more precision. Multivariable model: adjusted for age, sex, race/ethnicity, height, weight, education, income, neighborhood SES index, smoking status, pack-years, total cholesterol, HDL, hypertension, systolic blood pressure, fasting glucose, diabetes, C-reactive protein, left ventricular mass and city. P-interactions: sex 0.69, race/ethnicity 0.003, age group 0.18, smoking status 0.71, airflow limitation 0.28, emphysema 0.86.

Table S1: City-specific and overall correlations between $PM_{2.5}$ and NO_2 exposures

Study site	Ambient $PM_{2.5}$	Individually-weighted $PM_{2.5}$
**Forsyth County, NC**		
Ambient NO_2	0.59	0.37
**New York, NY**		
Ambient NO_2	0.57	0.49
**Baltimore, MD**		
Ambient NO_2	0.53	0.38
**St. Paul, MN**		
Ambient NO_2	0.81	0.55
**Chicago, IL**		
Ambient NO_2	0.58	0.32
**Los Angeles, CA**		
Ambient NO_2	0.67	0.43
**Overall**		
Ambient NO_2	0.67	0.77

All p-values < 0.001

Table S2: Mean differences in RV mass, end-diastolic volume, mass/end-diastolic volume ratio, stroke volume and ejection fraction adjusted for LV parameters per 5 μg/m^3 increase in ambient $PM_{2.5}$ exposure, stratified by city

RV parameter	Forsyth County, NC (n=598)	New York, NY (n=818)	Baltimore, MD (n=735)	St. Paul, MN (n=607)	Chicago, IL (n=556)	Los Angeles, CA (n=727)
**RV mass, g**						
Multivariable model	-0.84 (-2.20, 0.51)	-0.55 (-1.24, 0.15)	0.48 (-0.73, 1.69)	3.86 (2.56, 5.16)*	0.07 (-0.74, 0.89)	-0.65 (-1.34, 0.04)
**RV end-diastolic volume, mL**						
Multivariable model	-10.53 (-17.16, -3.90)*	-2.65 (-6.18, 0.88)	-2.86 (-9.27, 3.55)	6.20 (-0.12, 12.53)	-1.77 (-6.01, 2.47)	0.94 (-2.68, 4.56)
**RV mass/end-diastolic volume ratio, g/mL**						
Multivariable model	-0.002 (-0.01, 0.007)	0.001 (-0.003, 0.005)	0.002 (-0.005, 0.009)	0.017 (0.010, 0.024)*	0.005 (-0.001, 0.010)	-0.008 (-0.01, -0.003)*
**Stroke volume, mL**						
Multivariable model	-7.30 (-12.08, -2.52)*	-2.52 (-5.11, 0.07)	-1.14 (-5.71, 3.44)	2.27 (-2.77, 7.30)	-1.95 (-5.04, 1.14)	0.10 (-2.35, 2.55)
**RV ejection fraction, %**						
Multivariable model	-0.24 (-2.92, 2.45)	-0.46 (-1.70, 0.78)	1.55 (-0.47, 3.56)	-1.18 (-3.44, 1.07)	-0.95 (-2.48, 0.58)	-0.37 (-1.72, 0.97)

Multivariable model: adjusted for age, sex, race/ethnicity, height, weight, education, income, neighborhood SES index, smoking status, pack-years, total cholesterol, HDL, hypertension, systolic blood pressure, fasting glucose, diabetes, C-reactive protein and respective left ventricular parameter

*P-value < 0.05

P-interaction for city: RV mass <0.001, RV end-diastolic volume = 0.16, RV mass/end-diastolic volume ratio <0.001, Stroke volume = 0.13, RV ejection fraction = 0.75.

Table S3: Mean differences in RV mass, end-diastolic volume, mass/end-diastolic volume ratio, stroke volume and ejection fraction adjusted for LV parameters per 5 μg/m^3 increase in ambient $PM_{2.5}$ and individually-weighted $PM_{2.5}$ exposure, with a random effect for city.

RV parameter	Ambient $PM_{2.5}$ Estimate per 5 μg/m^3 (95% CI)	Individually-weighted $PM_{2.5}$ Estimate per 5 μg/m^3 (95% CI)
**RV mass, g**		
Multivariable model + city	0.32 (0.01, 0.63)*	0.29 (0.02, 0.56)*
**RV end-diastolic volume, mL**		
Multivariable model + city	-0.83 (-2.45, 0.78)	-0.14 (-1.56, 1.27)
**RV mass/EDV ratio, g/mL**		
Multivariable model + city	0.002 (0.001, 0.004)*	0.001 (-0.001, 0.002)
**Stroke volume, mL**		
Multivariable model + city	-0.93 (-2.09, 0.23)	0.09 (-0.95, 1.13)
**RV ejection fraction, %**		
Multivariable model + city	-0.04 (-0.15, 0.07)	0.08 (-0.40, 0.55)

Multivariable model: adjusted for age, sex, race/ethnicity, height, weight, education, income, neighborhood SES index, smoking status, pack-years, total cholesterol, HDL, hypertension, systolic blood pressure, fasting glucose, diabetes, C-reactive protein and respective left ventricular parameter

* P-value < 0.05

Table S4: Mean differences in RV mass, end-diastolic volume, mass/end-diastolic volume ratio, stroke volume and ejection fraction adjusted for LV parameters and NO_2 exposure per 5 μg/m^3 increase in ambient $PM_{2.5}$ (N=4,028) and individually-weighted $PM_{2.5}$ (N=3,368) exposure

RV parameter	Ambient $PM_{2.5}$ Estimate per 5 μg/m^3 (95% CI)	Individually-weighted $PM_{2.5}$ Estimate per 5 μg/m^3 (95% CI)
**RV mass, g**		
Multivariable model	-0.12 (-0.32, 0.09)	0.04 (-0.19, 0.26)
Multivariable model + city	0.09 (-0.34, 0.52)	0.21 (-0.06, 0.47)
**RV end-diastolic volume, mL**		
Multivariable model	-4.01 (-5.05, -2.96)*	-2.66 (-3.80, -1.51)*
Multivariable model + city	-2.53 (-4.74, -0.32)*	-0.25 (-1.89, 1.39)
**RV mass/EDV ratio, g/mL**		
Multivariable model	0.005 (0.004, 0.006)*	0.004 (0.002, 0.004)*
Multivariable model + city	0.002 (-0.001, 0.004)	0.0003 (-0.002, 0.002)
**RV Stroke volume, mL**		
Multivariable model	-3.39 (-4.14, -2.63)*	-2.10 (-2.94, -1.26)*
Multivariable model + city	-2.14 (-3.74, -0.54)*	0.02 (-1.18, 1.22)
**RV ejection fraction, %**		
Multivariable model	-0.45 (-0.81, -0.08)*	-0.23 (-0.64, 0.18)
Multivariable model + city	-0.17 (-0.95, 0.61)	0.14 (-0.45, 0.73)

Multivariable model: adjusted for age, sex, race/ethnicity, height, weight, education, income, neighborhood SES index, smoking status, pack-years, total cholesterol, HDL, hypertension, systolic blood pressure, fasting glucose, diabetes, C-reactive protein, respective left ventricular parameter and NO_2 exposure

* P-value < 0.05

Table S5: Characteristics of participants with ambient $PM_{2.5}$ measured and the subset with individually-weighted $PM_{2.5}$.

Characteristic	Ambient $PM_{2.5}$ (N=4,041)	Individually-weighted $PM_{2.5}$ (N=3,379)
**Age, years**	61.5	61.0
**Male, %**	47.6	47.8
**Race, %**		
White	39.0	39.2
Black	26.9	25.9
Hispanic	21.8	21.5
Chinese	12.3	13.4
**Height, cm**	166.4±9.9	166.6±9.9
**Weight, kg**	77.6±16.2	77.6±16.2
**Smoking, %**		
Never	46.8	47.8
Former	39.5	39.2
Current	13.7	13.1
**U.S. City, %**		
Forsyth County, North Carolina	14.8	14.7
New York, New York	20.2	21.3
Baltimore, Maryland	18.2	15.4
St. Paul, Minnesota	15.0	14.2
Chicago, Illinois	13.8	15.4
Los Angeles, California	18.0	19.1
**RV mass, g**	21.0±4.4	21.1±4.4
**RV end diastolic volume, mL**	124.0±30.8	125.0±30.75
**RV mass/end-diastolic volume ratio, g/mL**	0.17±0.02	0.17±0.02
**RV stroke volume, mL**	86.8±20.5	87.4±20.5
**RV ejection fraction, %**	70.5±6.4	70.4±6.4

Figure S1: Hypothesized causal diagram

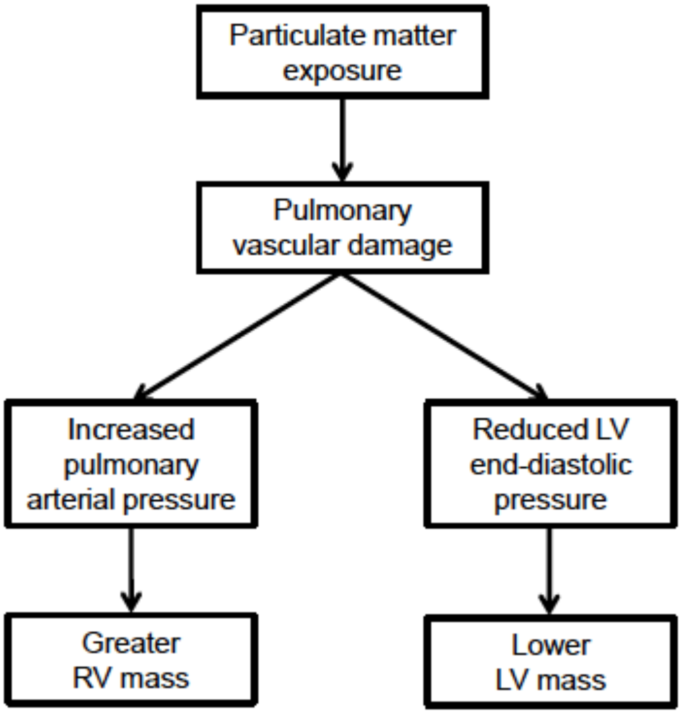

Figure S2: Description of the study sample

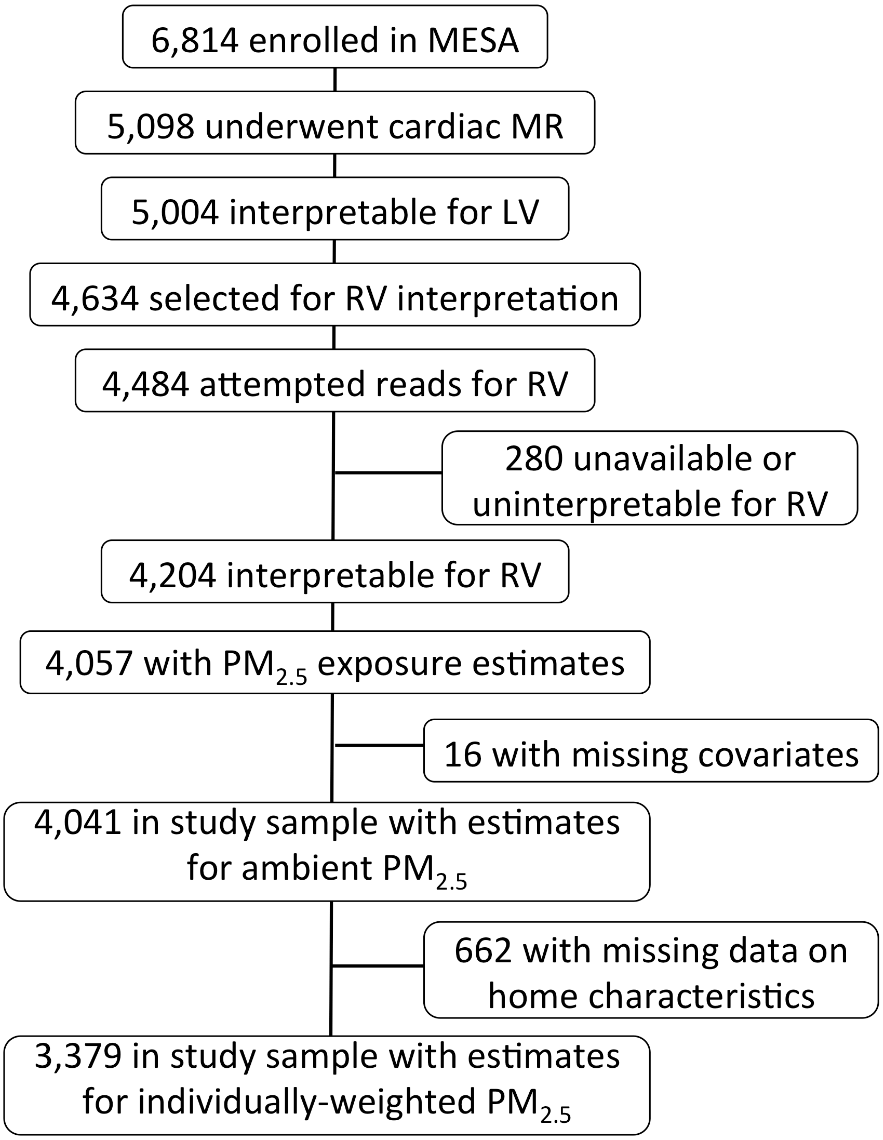

Figure S3: Sensitivity analyses for the multivariable association of individually-weighted $PM_{2.5}$ exposure and RV mass adjusted for LV mass and city

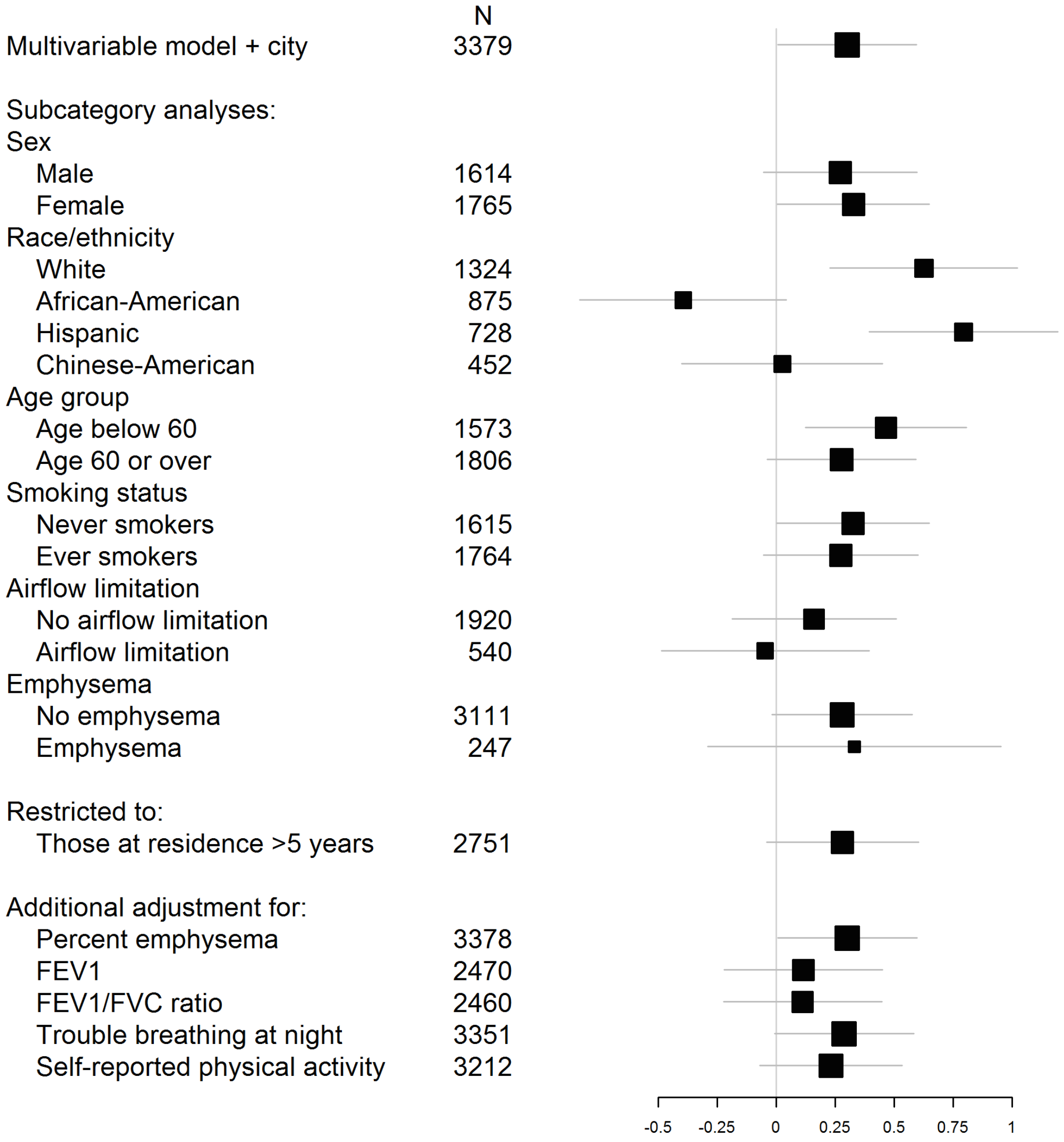

Shown are the mean differences (■) and 95% confidence limits for a 5 μg/m^3 change in $PM_{2.5}$. The size of the square reflects the relative precision of the estimate. Larger squares represent more precision. Multivariable model: adjusted for age, sex, race/ethnicity, height, weight, education, income, neighborhood SES index, smoking status, pack-years, total cholesterol, HDL, hypertension, systolic blood pressure, fasting glucose, diabetes, C-reactive protein, left ventricular mass and city. P-interactions: sex 0.69, race/ethnicity 0.003, age group 0.18, smoking status 0.71, airflow limitation 0.28, emphysema 0.86.
